# Evaluation of the contact surface between vertebral endplate and 3D printed patient-specific cage vs commercial cage

**DOI:** 10.1038/s41598-022-16895-9

**Published:** 2022-07-22

**Authors:** Renan Jose Rodrigues Fernandes, Aaron Gee, Andrew James Kanawati, Fawaz Siddiqi, Parham Rasoulinejad, Radovan Zdero, Christopher Stewart Bailey

**Affiliations:** 1 Combined Orthopaedic and Neurosurgery Spine Program, London Health Science Centre, Victoria Hospital, 800 Commissioners Road E (RM A6-144), London, ON N6A 5W9 Canada; 2 Schulich School of Medicine, Western University, London, ON Canada; 3 Lawson Health Research Institute, London, ON Canada; 4 Westmead Hospital, Sydney, NSW Australia

**Keywords:** Biomedical engineering, Engineering, Anatomy, Musculoskeletal system, Bone, Translational research

## Abstract

Biomechanical study. To evaluate the performance of the contact surface for 3D printed patient-specific cages using CT-scan 3D endplate reconstructions in comparison to the contact surface of commercial cages. Previous strategies to improve the surface of contact between the device and the endplate have been employed to attenuate the risk of cage subsidence. Patient-specific cages have been used to help, but only finite-element studies have evaluated the effectiveness of this approach. There is a possible mismatch between the CT-scan endplate image used to generate the cage and the real bony endplate anatomy that could limit the performance of the cages. A cadaveric model is used to investigate the possible mismatch between 3D printed patient-specific cages and the endplate and compare them to commercially available cages (Medtronic Fuse and Capstone). Contact area and contact stress were used as outcomes. When PS cage was compared to the Capstone cage, the mean contact area obtained was 100 ± 23.6 mm^2^ and 57.5 ± 13.7 mm^2^, respectively (p < 0.001). When compared to the Fuse cage, the mean contact area was 104.8 ± 39.6 mm^2^ and 55.2 ± 35.1 mm^2^, respectively(p < 0.001). Patient-specific cages improve the contact area between the implant and the endplate surface, reducing the contact stress and the risk of implant subsidence during LIF surgeries.

## Introduction

Lumbar interbody fusion (LIF) has been widely used to treat different pathologies in the lumbar spine, but several complications have been described^1,2^. Implant subsidence, defined when the cage penetrates one or both adjacent vertebral bodies' endplate, remains the most common challenge to overcome. It can cause loss of segmental lordosis, relapse of foraminal compression, instability, and pain^3^. Several strategies can be used to help reduce the incidence of subsidence, including the use of different cage materials, alternative surgical approaches allowing for the placement of larger cages, and increasing the total area of contact between the cage and the vertebral body endplate^4–6^.

Rapid prototyping (RP) is among the options to increase the contact area between the interbody fusion device and the endplate. It facilitates the development of a three-dimensional (3D) customized implant matching the patient's endplate bone anatomy, thus increasing the contact area when compared to commercially available implants^7,8^. This strategy can play a critical role in posterior approaches using posterior interbody fusion (PIF) cages since the small dissection window limits the size that can be placed inside the disc space.

Some papers have studied the effectiveness of conformational implants for LIF using Finite Element (FE) models^9–11^, but they have a perfect match of the cross-sectional area between the digital 3D bone model and the digital 3D cage model, which is not expected to happen in the clinical scenario because of endplate preparation and implant manufacturing limitations.

The settings used in the clinic computed tomography (CT) scans protocol may pose limitations to the customized implant model due to resolution restraint ^12^. Also, the 3D printer processing parameters such as the part orientation and layer thickness can influence the final resolution of the 3D printed device^13,14^. Digitally, the implant and bone model may match, but there would be a mismatch in the resulting 3D printed implant and actual bone surface, and the PS cage has to be placed in the exact same position which it has been planned digitally to be effective. Although intuitively, a patient-specific (PS) design should result in a higher area of contact, the actual significance is not known in the in vitro model only the less clinical finite element model.

The present study uses a cadaveric model to investigate the capacity of 3D printed patient-specific cages to increase the vertebral body endplate's contact area and compare their performance to commercially available cages.

## Materials and methods

After Research Ethics Board approval (Western University Health Research Ethics Board #115303), five full (n = 5) unidentified spine cadaveric specimens were purchased through a donor organization that provides cadavers for medical research and education. All the procedures performed during the study were in accordance with institutional guidelines and regulations for cadaveric and biomechanical studies, following recommended safety protocols. Informed consent authorizing the patient's body or cadaveric specimen to be used for educational, research and scientific purposes was obtained from next of kin by the donor organization.

The specimens were subject to CT-Scan to rule out bone tumours or fractures and obtain image acquisition for the segmentation process. The specimens were kept frozen and soft tissues remained intact during the scanning process to replicate the clinical scenario as much as possible.

**Figure 1:**
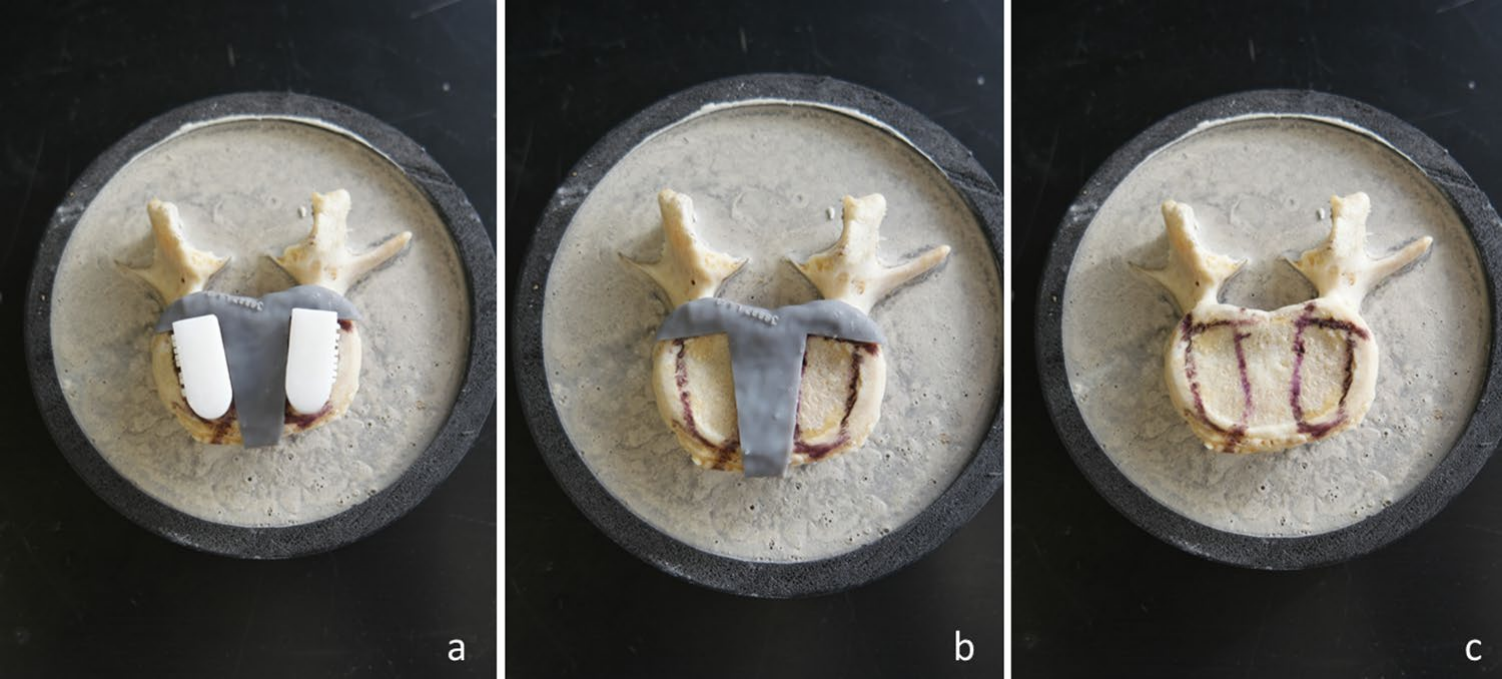
L5 vertebra potted in cement for biomechanical testing.

After, the spines were isolated from L1 to L5 and prepared in a similar way, as described in previous studies^15^. A combination of gentle sharp dissection using a scalpel, curettes, and periosteal elevators were used to remove soft tissue. We took special care during the cartilaginous endplate removal to avoid damage to the underlying bony endplate. After removing the soft tissue, the bones dried at room temperature and were potted in cement with the cranial endplate parallel to the ground (Fig. 1).

### Bone segmentation

An open-source software, 3D Slicer (version 4.10.2), was used to create the vertebrae 3D mesh models by importing the CT digital imaging and communication in medicine (DICOM) files.

**Figure 2:**
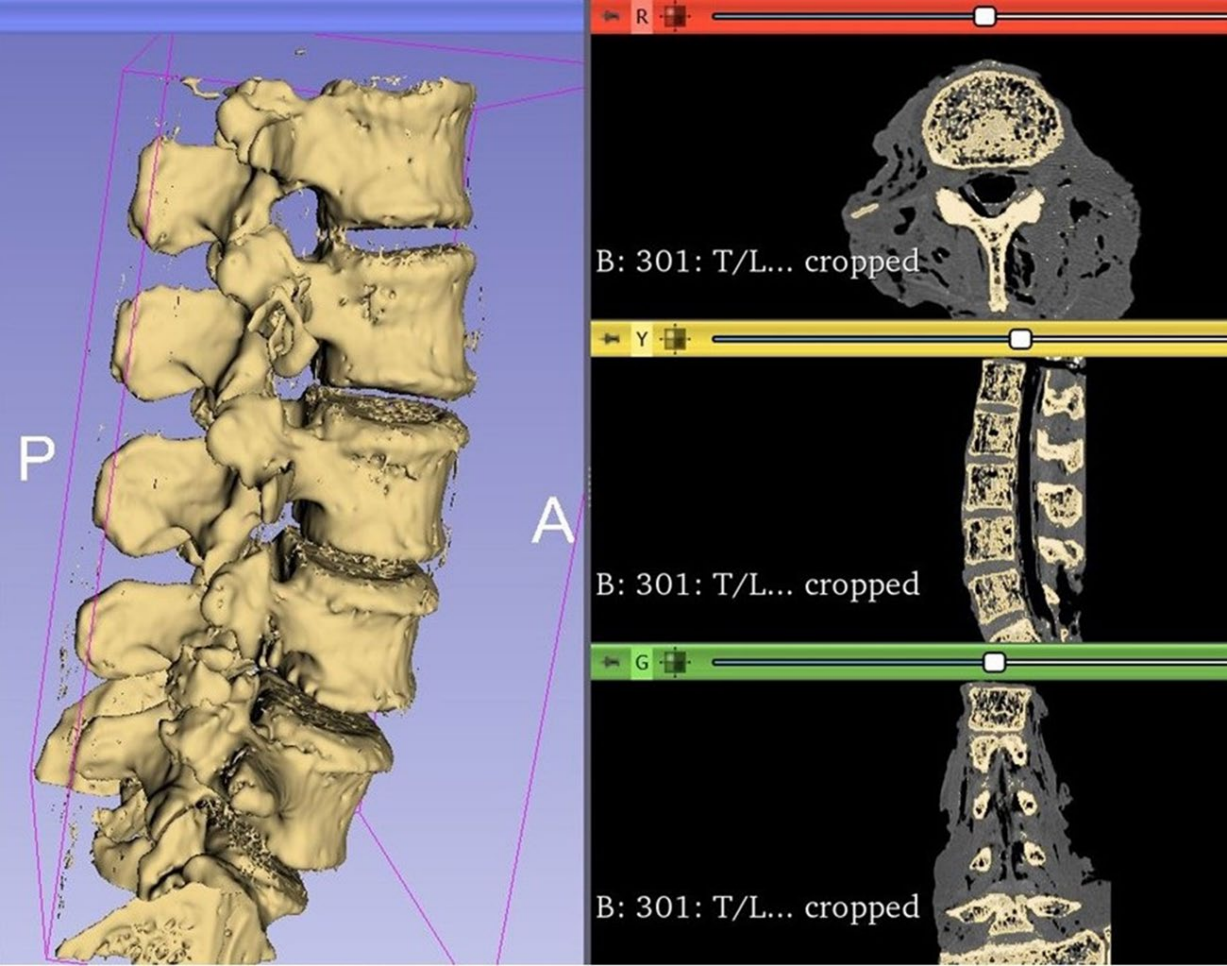
Example of 3D segmentation of the lumbar spine using 3D Slicer. Each vertebra file was saved as an individual file.

A region of interest was created around the lumbar spine (L1–L5), using the data from the 0.625 mm slice thickness bone reconstructions. Each endplate model was created using semi-automatic segmentation by the 'grow from seeds' extension in the Segment Editor of 3D Slicer (Fig. 2). Bone and soft tissue were extracted based on different Hounsfield Units (HU). Segmentation defects were corrected by modifying seeds when needed, with care taken to compare the final segmentation model to the original CT-scan reconstruction. Also, a 2 mm external shell mirroring the vertebrae's contour was created to latter allow them to be used as guides to place the cages in the desired position during testing (Fig. 1).

### Choice of commercially available LIF cage models

**Figure 3:**
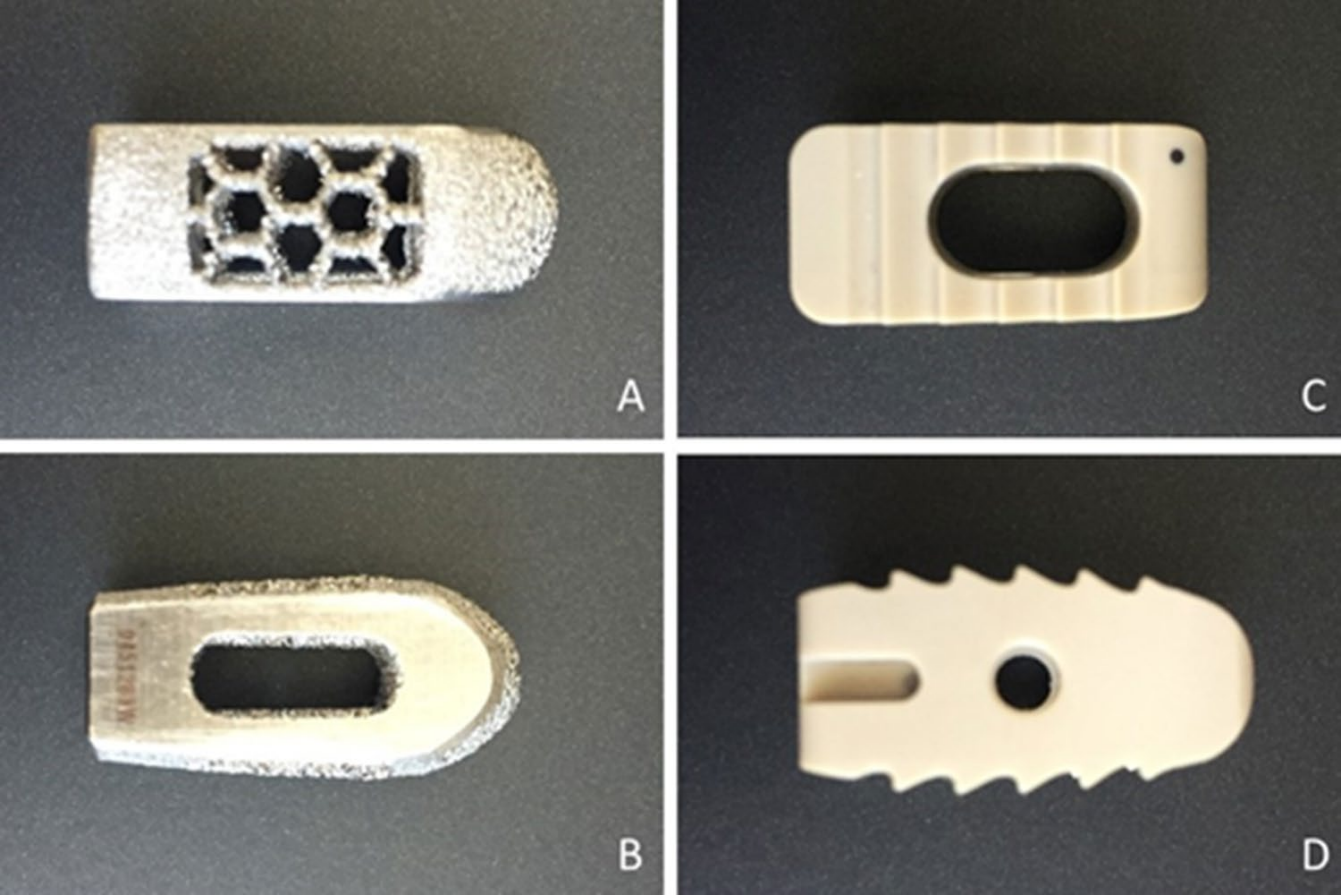
Superior and lateral views of Fuse cage (**A,B**) and Capstone cage (**C,D**).

We used two types of commercially available intervertebral LIF cages provided by a single prominent medical device company supplier (Medtronic Sofamor Danek USA, Inc., Memphis, TN, USA). One was made out of titanium alloy and had a cylindrical shape (Fuse Spinal System); the other was made out of PEEK and had a rectangular shape (Capstone PEEK Spinal System) (Fig. 3).

**Figure 4:**
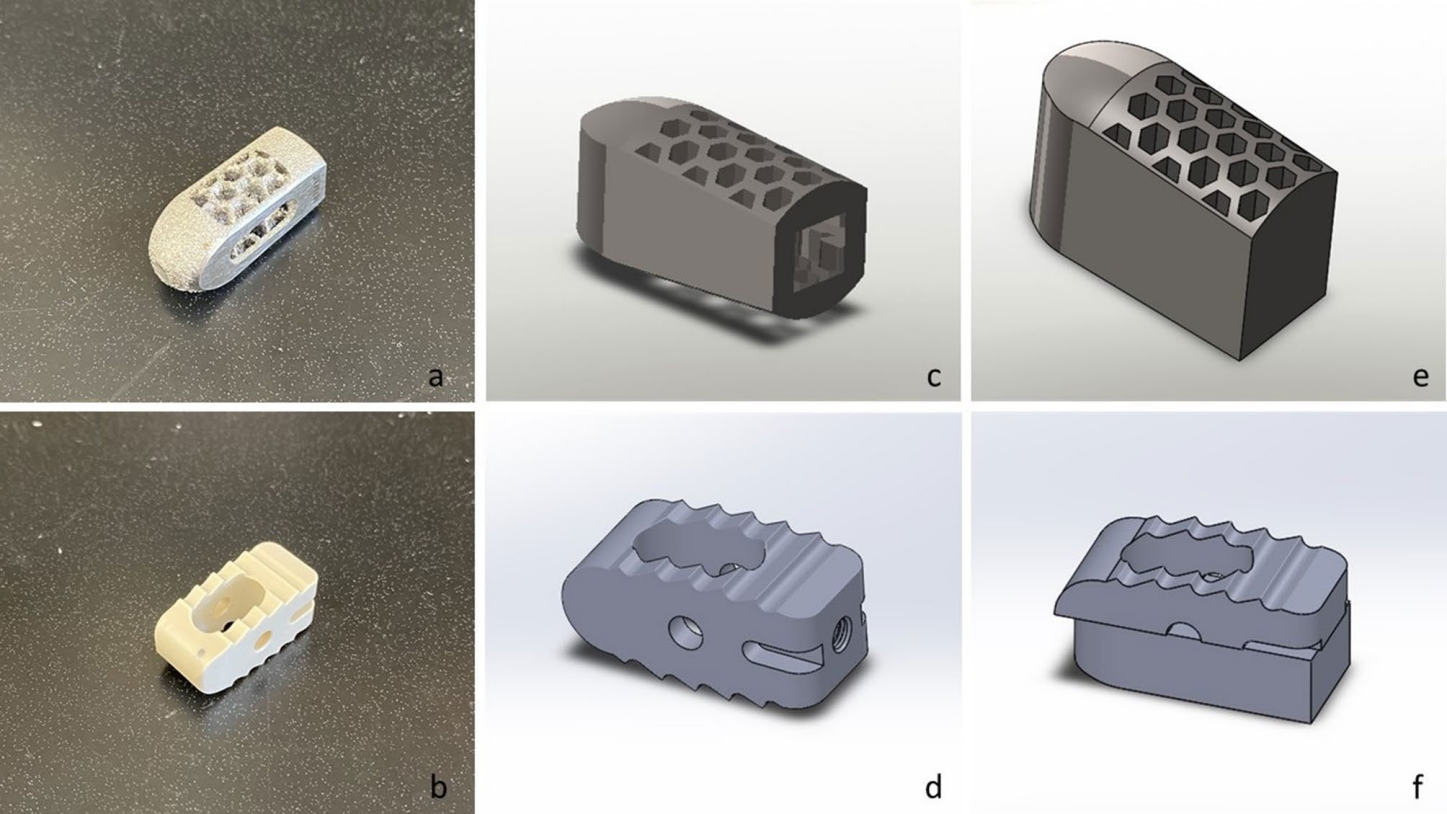
Images showing pictures of the original Fuse and Capstone cages (**a,b**), their full implant CAD models (**c,d**), and the CAD models used for the biomechanical testing (**f,g**).

Reverse engineering processes were used to replicate the dimensions and features of the commercial cages. The modified implant models for subsidence testing were designed in CAD 3D modelling software (SolidWorks 2019, Dassault Systemes Solidworks Corp.). They were initially designed as a full implant and then cut in half to allow the addition of a base of support to enable the implant to be attached to the testing machine (Fig. 4), and the cylindrical cage model and the rectangular cage model corresponding files were exported as STL file.

### Patient-specific cages

**Figure 5:**
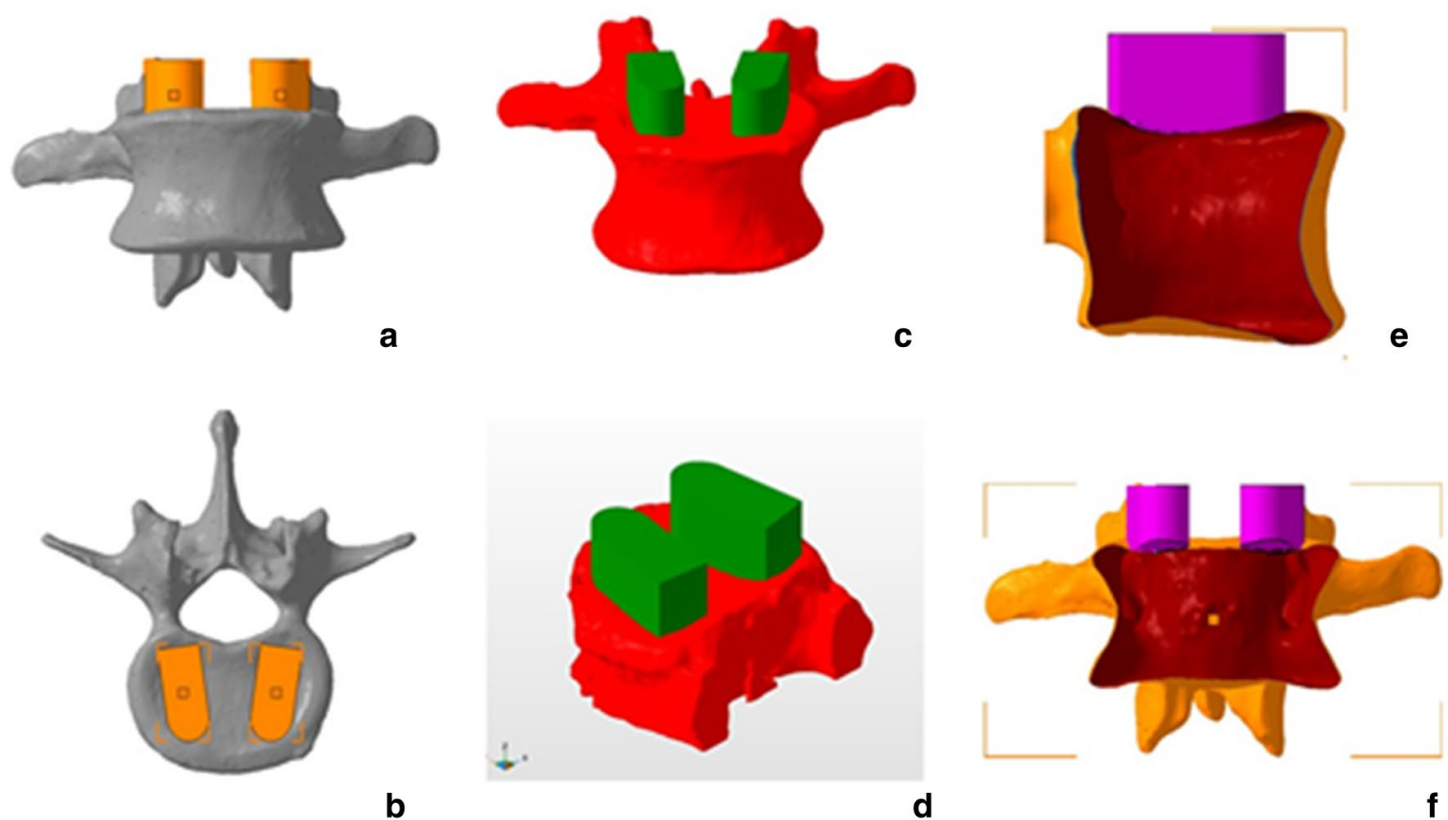
Anterior and superior views of cage planned positions(**a,b**), anterior and oblique views of the Boolean operation(**c,d**), and lateral and anterior views of the hollow vertebra and the conformational implant post-Boolean operation (**e,f**).

The vertebrae models and the cylindrical cage model were imported into STL editing software (Netfabb, Autodesk Inc, San Rafael, California). Two replicated commercial implants (left and right) were translated for every endplate until their geometry was overlapping with each vertebra's superior endplate in a similar position it would be placed during a LIF surgery, thus determining the area where they would be tested in the cadaveric bone. After that, a Boolean subtraction operation was performed to create two patient-specific LIF cages per vertebra and the endplate guides (Figs. 1 and 5).

### Cage 3D printing

All the STL files, including the patient-specific cages, and the modified cylindrical and rectangular-shaped cages, were imported into the FormLabs PreForm software to be printed using a Form 2 printer (FormLabs, Somerville, Massachusetts). The layer thickness was set to 50 microns to improve resolution. To standardize the material, all the models were printed in Rigid resin (FormLabs, Somerville, Massachusetts) reinforced with glass fibre making it resistant to deformation and having an Elastic Modulus similar to PEEK.

Eighteen of the available 25 vertebrae were utilized for testing, a sample size similar to previously published papers using pressure-sensitive film^16,17^. Four vertebrae were excluded from the 25 dissected lumbar vertebrae because they had been damaged during the cadaver harvesting process. Another three vertebrae were excluded, one due to a previous fracture, another due to Schmorl's nodes and a third one was damaged during potting. The 18 vertebrae were then split into two groups of 9 vertebrae each: group 1 compared patient-specific vs. replicated Capstone, and group 2 compared patient-specific vs. replicated Fuse. Each vertebra's left and the right side was tested for both the patient-specific cage and the replicated commercial cage allowing 18 samples per comparison group.

### Testing set-up

**Figure 6:**
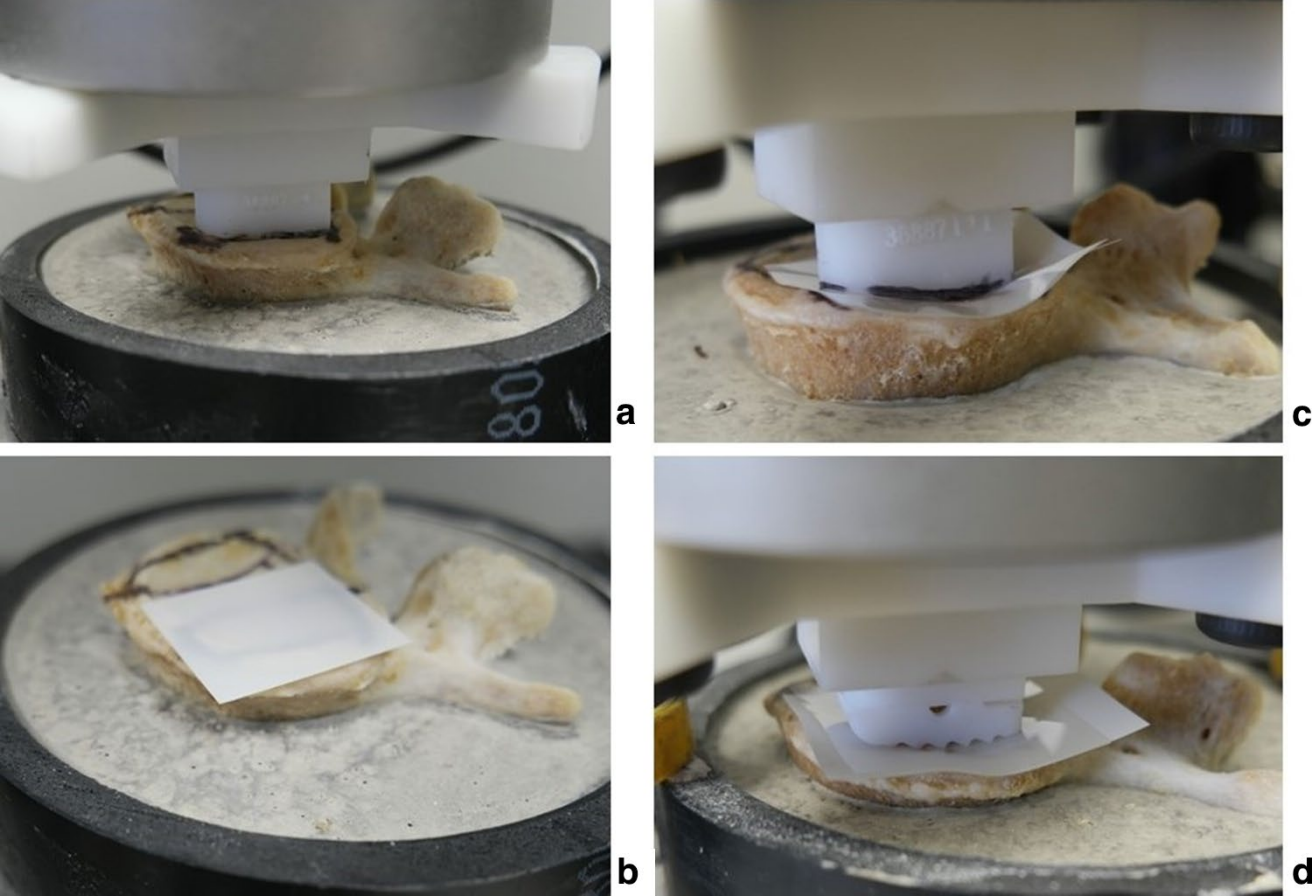
(**a**) Ideal cage position was determined using PS cage, (**b**) FujiFilm was put in place, (**c,d**) PS and commercial cage being compressed over the FujiFilm.

Pressure-sensitive measurement film ("Ultra super low" Fujifilm, Pressure Metrics, Whitehouse Station, NJ, USA) was inserted at the interface of each cage-vertebra construct. The sheets were cut into 30 mm × 30 mm squares. The Fujifilm indicator layer was placed on the top of the endplate, and the acid layer above the indicator layer (Fig. 6). 3D printed guides were used to determine the ideal cage position (Fig. 1). Using an electromechanical testing machine (Instron 5967, Norwood, MA, USA), the cages were compressed axially over the vertebra's endplate with a 100 Newtons(N) force for 30 s to obtain a consistent stain. The 100 N force was chosen to avoid damage to the endplate. All cages were packed with bone graft to replicate similar conditions in surgery. Finally, the endplate surface was thoroughly inspected after every test to assess any surface conditioning that could interfere with the next test.

### Contact area and contact stress analysis

**Figure 7:**
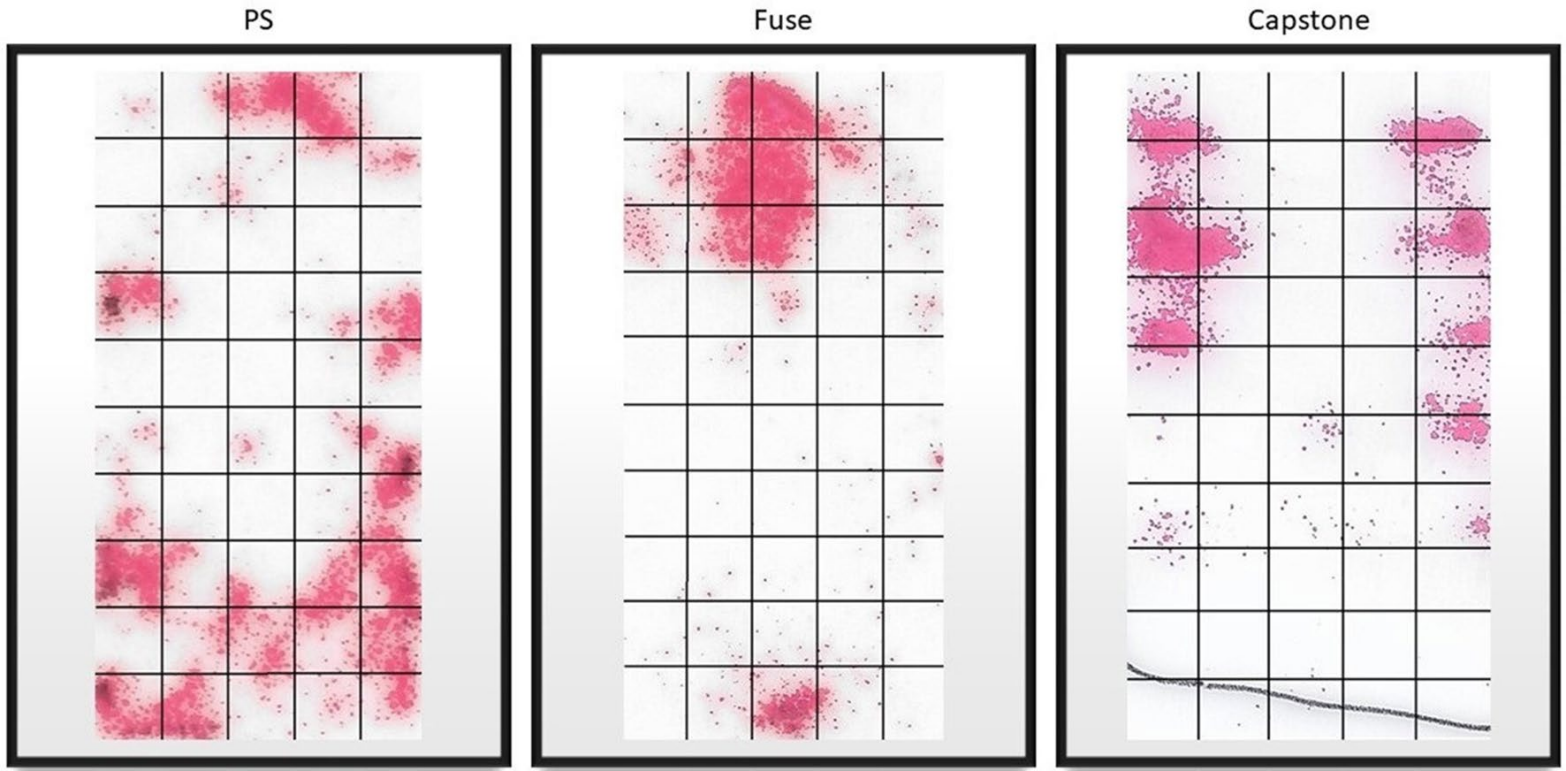
Sample imaging of the contact area for each of the cages.

After the load was removed, Fujifilm sheets were carefully removed from the top of the endplate. Afterward, the Fujifilm indicator layers' contact areas were scanned in jpeg format at 1200 dpi (dots per inch) using a desktop scanner (Hewlett-Packard, HP ENVY 4520) (Fig. 7). The maximum contact area of the cages that were touching the endplates was calculated using the ImageJ software (version 1.52, U. S. National Institutes of Health, Bethesda, Maryland, USA). Mean contact stress was obtained by dividing the applied force (100 N) by the measured contact area and was reported in megapascals (MPa).

### Statistical analysis

All statistical analyses were conducted using IBM SPSS version 26 (IBM Corp., Armonk, New York, USA). Comparison between cages in each group was made using the Mann–Whitney U test. Statistical significance was set at p < 0.05.

### Research ethics approval

#115303, Western University Health Research Ethics Board.

## Results

### Cage contact area

In group 1, in which the PS cage was compared to the replicated Capstone cage, the mean contact area obtained was 100 ± 23.6 mm^2^ and 57.5 ± 13.7 mm^2^, respectively. In group 2, the PS cage was compared to the replicated Fuse cage; the mean contact area was 104.8 ± 39.6 mm^2^ and 55.2 ± 35.1 mm^2^, respectively. For both groups, the PS mean contact area was significantly different than the Capstone and Fuse cages' contact areas (p < 0.001) (Table 1).

**Table 1:** Mean ± SD cage contact areas, in mm^2^, and contact stress, in megapascals (MPa).

Group	Cage	N	Mean	SD	P
**Contact area**					
Group 1	Patient-specific	18	100	23.6	< 0.001
	Capstone	18	57.5	13.7	
Group 2	Patient-specific	18	104.8	39.6	< 0.001
	Fuse	18	55.2	35.1	
**Contact stress**					
Group 1	Patient-specific	18	1.06	0.28	< 0.001
	Capstone	18	1.84	0.45	
Group 2	Patient-specific	18	1.10	0.43	< 0.001
	Fuse	18	2.44	1.32	

### Contact stress

In group 1, the Capstone cage mean contact stress was 73% higher than the PS cage (1.84 vs. 1.06 MPa, p < 0.001)), while in group 2, the Fuse cage mean contact stress was 122% higher than the PS cage (2.44 vs. 1.10 MPa, p < 0.001).

### Contact footprint vs total surface area

Each cage had a theoretical maximum contact area of 220mm^2^. Fujifilm analysis shows that PS cages combined had an average contact footprint of 46.5% of the total area (mean = 102.4mm^2^). The Capstone and Fuse cages had a contact footprint of 26.1% and 25.1% of the total possible area, respectively.

## Discussion

This study found that LIF patient-specific cages increased the contact area between the cage and the endplate by up to 74% compared to commercially available LIF cages. It resulted in better utilization of the cage's total area with better load sharing across the endplate and, therefore, resulted in significantly lower contact stress.

A biomechanical study^6^ showed that by increasing the cage's size, the force required for subsidence increases due to a larger contact area, but they found out that when only the length of LIF cages was increased, it did not increase substantially the force required for subsidence. It was attributed to the cage design since the contact area was restricted to the cage indentations no matter the length of the cage.

Analyzing contact area and pressure is essential to understanding medical devices' biomechanical behaviour and their interaction with the human bone. Knowledge of this interaction can provide metrics to improve future medical device design. When it comes to developing patient-specific interbody fusion devices, studies have explored finite element models to evaluate stress distribution across the endplate^9–11^. They found that devices matching the endplate surface anatomy promote a reduction in the endplate stresses and subsidence risk. Due to the natural concave shape of the endplate, Patel^10^ showed that for non-conformational implants, high-stress concentrations were located at the edges of the endplate-device interface, supporting our findings for the commercial cages' contact analysis.

In contrast to a finite element patient-specific model that assumes a perfect match to the endplate contour, our 3D printed patient-specific cages did not have the same effectiveness displaying the real world challenges in fabricating an implant achieving a perfect match that is not apparent in computer models. None of the tested PS cages stained 100% of the cage area during the Fujifilm compression test. However, even without 100% contact area, results still demonstrated that an implant utilizing patient-specific geometry yielded significantly more stain area resulting in lower contact stresses using patient specific-cages compared to commercial cages. The lack of a complete match between the cage and the endplate is likely related to the CT scan's limitations. Image quality is determined by the choice of image parameters^12^. In this study, to evaluate the mismatch between the cage developed by the CT 3D vertebra reconstruction, a typical clinical lumbar spine image acquisition protocol was used. Kanawati et al. ^18^ compared 3D CT-based vertebrae models to 3D scanned cadaveric bones and found an excellent geometric overlap between models. Still, the CT 3D model had a significantly higher volume than the bone, showing a mismatch between the CT image and bone size. Our literature review did not identify a study that investigated the possible differences between the endplate CT image resolution and the bony endplate contour.

Nonetheless, there are limitations to this study. First, pressure-sensitive films are widely used for orthopedic biomechanics research, but they have several limitations related to handling, image processing, temperature and moisture sensitivity, and pressure thresholding^19^. For this study, an "Ultra super low" Fujifilm was used, and the pressure range was from 0.19 to 0.6 MPa. Since the force applied to the cages was limited to 100 N to avoid damage to the endplate during testing, the total contact area can be underestimated since the minimum threshold may not have been reached in some contact points. At the same time, folds on the film during the test can generate false-positive contact areas. Also, due to the low pressure range of the Fujifilm the stain's intensity was uniform at its max capacity and therefore stress distributions or high pressure regions could not be differentiated during image processing. It was only reported as the presence or absence of contact.

Secondly, this is an in-vitro cadaveric study; therefore, the meticulous process involved in cleaning the endplate may not be reproduced in-vivo during surgery and can create distortions in the final endplate aspect. Moreover, the 3D printing process itself can add errors to the cage's final aspect since the mere change in the device orientation in the printing platform can cause distortions related to the resin curing process. FormLabs Rigid resin was used to print all the cages in this study because it has an Elastic Modulus similar to PEEK's Elastic Modulus being resistant to deformation when subjected to high forces and more practical for laboratory tests. Also, placing the cages in the ideal position during testing to improve performance would not be possible in the clinical scenario without imaging support (e.g., intraoperative navigation).

Despite the limitations mentioned above, the present study proposed investigating whether small 3D printed patient-specific devices, such as LIF cages inserted from a posterior approach, reflect an increase in the contact area similar to that described by the computational models of endplate and implant contact. The study demonstrated the presence of a mismatch between the CT-based 3D vertebra model and the real endplate anatomy. Still, LIF patient-specific cages can achieve a larger contact area than "one-size-fits-all" commercially available cages.

## Conclusion

As shown in the present study, patient-specific cages used in a controlled environment can maximize the contact area between the implant and the endplate surface, reducing the contact stress and the risk of implant subsidence during LIF surgeries. The limitations posed by the CT-scan and 3D printer resolution used in our study affected the performance of the cages but improvements in medical imaging modalities and 3D printing devices can break down this barrier.

## Data Availability

The datasets generated and/or analysed during the current study are not publicly available due to ethical restrictions but are available from the corresponding author on reasonable request.
